# Sero-Diagnosis of *Mycobacterium avium* Complex Lung Disease Using Serum Immunoglobulin A Antibody against Glycopeptidolipid Antigen in Taiwan

**DOI:** 10.1371/journal.pone.0080473

**Published:** 2013-11-18

**Authors:** Chin-Chung Shu, Manabu Ato, Jann-Tay Wang, Ruwen Jou, Jann-Yuan Wang, Kazuo Kobayashi, Hsin-Chih Lai, Chong-Jen Yu, Li-Na Lee, Kwen-Tay Luh

**Affiliations:** 1 Graduate Institute of Clinical Medicine, College of Medicine, National Taiwan University, Taipei, Taiwan; 2 Department of Traumatology, National Taiwan University Hospital, Taipei, Taiwan; 3 Department of Internal Medicine, National Taiwan University Hospital, Taipei, Taiwan; 4 Department of Immunology, National Infectious Diseases, Tokyo, Japan; 5 Center for Research, Diagnostics and Vaccine Development, Centers for Disease Control, Taipei, Taiwan; 6 Department of Medicine, Asoka Hospital, Tokyo, Japan; 7 Department of Medical Biotechnology and Laboratory Science, Chang Gung University, Tao-Yuan, Taiwan; 8 Department of Laboratory Medicine, National Taiwan University Hospital, Taipei, Taiwan; University of Padova, Medical School, Italy

## Abstract

**Background:**

Lung disease (LD) due to non-tuberculous mycobacteria is an important clinical concern. Mycobacterium avium complex (MAC) is one of the most common causative agents but the diagnosis of MAC-LD remains challenging. Detection of serum IgA antibody against MAC glycopeptidolipid (GPL) has recently been shown to improve the diagnosis of MAC-LD, but has yet to be validated worldwide.

**Methods:**

This prospective study was conducted in a tertiary referral center in northern Taiwan and enrolled patients with MAC-LD, MAC contamination, other lung diseases, and control subjects. Serum immunoglobulin A (IgA) antibody against MAC-GPL was detected in the participants and its specificity and sensitivity was assessed.

**Results:**

There were 56 patients with MAC-LD, 11 with MAC contamination, 13 *M. kansasii*-LD, 26 LD due to rapidly-growing mycobacteria (RGM), 48 pulmonary tuberculosis, and 42 household contacts of patients with TB. Patients with MAC-LD were older and 32% of them had an underlying co-morbidity. By logistic regression, serum MAC-GPL IgA level was an independent predictor of MAC-LD among the study subjects and those with culture-positive specimens for MAC. By the receiver operating characteristic curve, serum MAC-GPL IgA had a good power to discriminate MAC-LD from MAC contamination. Under the optimal cut-off value of 0.73 U/mL, its sensitivity and specificity were 60% and 91%, respectively. Among MAC-LD patients, presence of co-morbidity was associated with MAC-GPL <0.73 U/ml in logistic regression analysis.

**Conclusions:**

Measurement of serum anti-MAC-GPL IgA level is useful for the diagnosis of MAC-LD. However, its implement in clinical practice for immuno-compromised hosts needs careful consideration.

## Introduction

Lung disease (LD) due to non-tuberculous mycobacteria (NTM) is an important clinical concern because the incidence and prevalence of NTM diseases have increased over the last ten years [1,2]. According to a study in Taiwan, a tuberculosis (TB) endemic area, NTM-LD increased from 1.26 to 7.94 per 100,000 in-patients per year from 2000 to 2008 [2]. Among the NTM diseases in Asia, Mycobacterium avium complex (MAC) is the most common cause of NTM-LD [2,3]. 

The diagnosis of MAC-LD is complicated because unlike *Mycobacterium tuberculosis*, MAC contamination of clinical specimens can come from environmental sources such as water, dust, and soil. It may colonize the respiratory tract without any accompanying invasive disease [4]. Thus, isolation of MAC from sputum is insufficient to document pulmonary infection. Previous studies have shown that the presence of MAC has a clinical relevance of around 35-42% [5,6]. According to guidelines of the American Thoracic Society (ATS) [1], the diagnosis of pulmonary disease due to Mycobacterium avium complex (MAC) is based on clinical, radiographic, and mycobacterial criteria. Thus, the entire process is complicated and time consuming, requiring clinical findings and repeated MAC isolation/culture from sputum. 

Moreover, it is very difficult to discriminate between MAC contamination and MAC-LD [7,8], or between MAC-LD and other mycobacterial infections without culture results. Clinical features such as symptomatic or radiographic findings are very similar in mycobacterial diseases. From the standpoint of infection control, it is particularly important to distinguish between MAC-LD and pulmonary tuberculosis (TB). Therefore, tests that can rapidly and accurately differentiate between MAC-LD and MAC contamination are needed. 

Among sero-diagnostic methods, detection of serum immunoglobulin A (IgA) antibody against MAC glycopeptidolipid (GPL), an important antigenic component of MAC bacterial wall, has been recently developed. It shows good diagnostic performance in Japan (MAC-GPL IgA, Tauns Laboratory Inc., Shizuoka, Japan) [9,10]. However, a study conducted in the United States reveals that the test is specific but less sensitive (70%) when the same cut-off value (0.7 U/mL) is used, despite sufficient sensitivity (80%) obtained at 0.3 U/mL as the cut-off value [11]. The studies in Japan have included immunocompetent patients, but a small number of immunocompromised patients such as lung cancer, and did not examined patients with diseases due to rapidly growing mycobacteria (RGM) possessing glycopeptidolipid antigen such as *M. abscessus*, *M. fortuitum*, and *M. chelonae* [9,10]. Therefore, the sero-diagnostic test requires validation in different ethnicities and geographic locations with variable background prevalence of *M. tuberculosis* and NTM. This study examined the clinical availability of the sero-diagnostic test for MAC-GPL IgA, including sensitivity and specificity, in Taiwan.

## Materials and Methods

### Patient enrollment

This prospective study was conducted at National Taiwan University Hospital in Taipei from January 2011 to January 2013. The hospital’s research ethics committee approved the study and all participants provided written informed consent. Patients aged >20 years and had respiratory sample(s) culture-positive for NTM were identified. Among them, we enrolled patients with MAC-LD, MAC contamination, rapidly growing mycobacteria (RGM, including *M. abscessus*, *M. fortuitum*, and *M. chelonae*)-LD or *M. kansasii*-LD diagnosed according to the ATS diagnostic guideline [1]. Patients with culture-proven pulmonary TB and their household contacts who had a result of interferon-gamma release assay (IGRA) but no clinical symptoms and radiographic findings suggestive for active pulmonary disease in the last 6 months were also enrolled. In this study, IGRA was performed by using T-SPOT.*TB* (Oxford Immunotech Ltd, Oxford, UK) [12]

Mycobacterial culture and species identification was performed as previously described [13]. Patients with human immunodeficiency virus (HIV) infection and bleeding tendency that increased the risk of blood sampling were excluded. 

### MAC-GPL IgA antibody measurement

All samples were stored in -20°C and examined in random order by a technician blinded to the patients’ clinical diagnoses. Serum MAC-GPL IgA was measured by an enzyme immunoassay kit (Tauns Laboratory Inc., Shizuoka, Japan) according to the manufacturer’s instructions. Data was expressed as U/mL in reference to standard curve using positive controls.

### Data collection

Clinical data, including age, sex, co-morbidities, history of pulmonary TB, and laboratory data at enrollment was recorded in a standardized case report form. Disease duration was defined as the period between the date of the first confirmed positive culture and the date of patient enrollment with NTM-LD and pulmonary TB. Typical histology of NTM infection included granulomatous inflammation and/or presence of acid fast bacilli [1]. Chest imaging was interpreted as noted in a previous study [3]. Radiographic patterns of the main pulmonary lesion were categorized as consolidative, fibro-cavitary, or nodular-bronchiectatic.

### Statistical analysis

Inter-group differences were analyzed by the student *t* test or Mann-Whitney *U* test for numerical variables, and chi-square test or Fisher’s exact test for categorical variables, as appropriate. Multivariate logistic regression analysis was applied to identify factors associated with MAC-LD. In the stepwise variable selection procedure, all potential predictors were included. Statistical significance was set at a two-sided *p*<0.05. 

The discriminative power of each significant predictor between MAC-LD and other groups or MAC contamination was compared using the receiver operating characteristic (ROC) curve and the area under the curve (AUC). The optimal cut-off value, defined as the one with the least (1 - sensitivity)^2^ + (1 - specificity), was used to calculate sensitivity and specificity [14]. All analyses were performed using the SPSS (Version 15.0, Chicago, IL).

## Results

The study enrolled 56 patients with MAC-LD, 11 with MAC contamination, 14 with *M. kansasii*-LD, 26 with RGM-LD (14 *M. abscessus*, 7 *M. fortuitum*, and 5 *M. chelonae*), 48 with pulmonary TB, and 42 TB household contacts. Among the 42 household contacts, 22 (19 without underlying disease) were IGRA-positive and the remaining 20 (18 without underlying disease) were IGRA-negative. 

Among the MAC-LD patients, the mean age was 67.8 years, 50% were male, and 32% had co-morbidity (Table 1). Seven MAC-LD patients had malignancies, including two with lung cancer and one each with buccal cancer, leukemia, hepatoma, cholangiocarcinoma, and bladder cancer. Among the 56 MAC-LD patients, respiratory specimens were smear-positive for acid-fast bacilli in 33 (59%), including 10 with high-grade (3+~4+) positivity. Fifty one MAC-LD patients had ≥2 sputum samples culture-positive for MAC (average, 5.2 sets [SD: 2.8]). In five MAC-LD patients with only one positive sputum culture, four had typical lung histology and the remaining one had one bronchial washing culture positive for MAC. Based on the radiographic pattern, six (11%), nine (16%), and 41 (73%) were classified as consolidative, fibro-cavitary, and nodular-bronchiectatic lung diseases, respectively.

**Table 1:** The participants’ clinical characteristics based on lung disease status.

	MAC-LD n=56	MAC contamination n=11	*M. kansasii*-LD n=14	RGM-LD** n=26	PTB n=48	TB contacts n=42
Age (years)	67.8 [16.0]	60.9 [18.5]	56.9 [17.4]*	67.0 [13.3]	60.9 [17.9]*	50.1 [18.5]*
Male gender	28 (50%)	7 (64%)	11 (79%)	16 (62%)	32 (67%)	13 (31%)
Disease duration (months)	13.2 [21.8]	-	2.9 [8.2]*	4.3 [7.5]*	1.3 [1.1]*	-
Underlying co-morbidity	18 (32%)	4 (36%)	3 (21%)	9 (35%)	23 (48%)	5 (12%)*
Diabetes mellitus	4 (7%)	1 (9%)	1 (7%)	3 (12%)	7 (15%)	3 (7%)
Malignancy***	7 (13%)	1 (9%)	1 (7%)	2 (8%)	9 (19%)	2 (5%)
Hemodialysis	0	1 (9%)*	0	2 (8%)*	3 (6%)	0
Autoimmune diseases****	5 (9%)	0	1 (7%)	0	2 (4%)	0*
Liver cirrhosis	2 (4%)	1 (9%)	0	2 (8%)	8 (17%)*	0
Past TB history	8 (14%)	2 (18%)	4 (29%)	1 (4%)	0*	0*
MAC GPL antibody, U/mL	4.27 [6.32]	0.45 [0.73]*	0.16 [0.15]*	1.11 [2.27]*	0.78 [2.47]*	0.28 [0.74]*
Leukocytes, per µL	6926 [2743]	7045 [2225]	9063 [5082]	7528 [3628]	7661 [3658]	-
Hemoglobin, g/dL	12.6 [2.1]	12.6 [2.5]	12.2 [2.3]	12.2 [2.2]	12.7 [2.0]	-

Abbreviations: GPL, glycopeptidolipid; LD, lung disease; MAC, *Mycobacterium avium* complex; NTM, non-tuberculous mycobacteria; PTB, pulmonary tuberculosis; RGM, rapidly-growing mycobacteria

Data are no. (%) or mean [standard deviation]

* *p*<0.05 between the indicated group and the MAC-LD group

** RGM includes 14 *M. abscessus*, 7 *M. fortuitum*, and 5 *M. chelonae*

*** Malignancy includes 5 lung cancer, 4 head and neck cancer, 3 leukemia, 3 hepatoma, 2 breast cancer, 2 bladder cancer, 2 biliary tract cancer, and 1 thyroid cancer

Autoimmune diseases includes 3 rheumatoid arthritis, and each one of systemic lupus erythematosus, ankylosing spondilytis, Sjögren syndrome, Graves' disease, and autoimmune related nephrotic syndrome

Among patients with MAC contamination, the mean age was 60.9 years and 64% were male. All had ≥3 sets of sputum for acid-fast smear and mycobacterial culture. Except for one who had three culture-positive sputum samples but normal chest radiography, all of the patients had only one culture-positive sputum sample. Two of the 11 persons with MAC contamination had low-grade positive acid-fast smear. 

Patients with MAC-LD were older than those with *M. kansasii*-LD, pulmonary TB, and TB contacts (Table 1). Disease duration was 13.2 months in average in the MAC-LD group and this was longer than those in the *M. Kansasii*-LD, RGM-LD, and pulmonary TB groups. In the MAC-LD group, 24 patients (42%) had disease duration less than 3 months, including 10 who were started on anti-MAC treatment upon enrollment. Prior TB history was more common in the NTM-LD group than in the PTB and control groups. Laboratory data, including blood hemoglobin and leukocyte count, were similar among all groups. 

**Figure 1:**
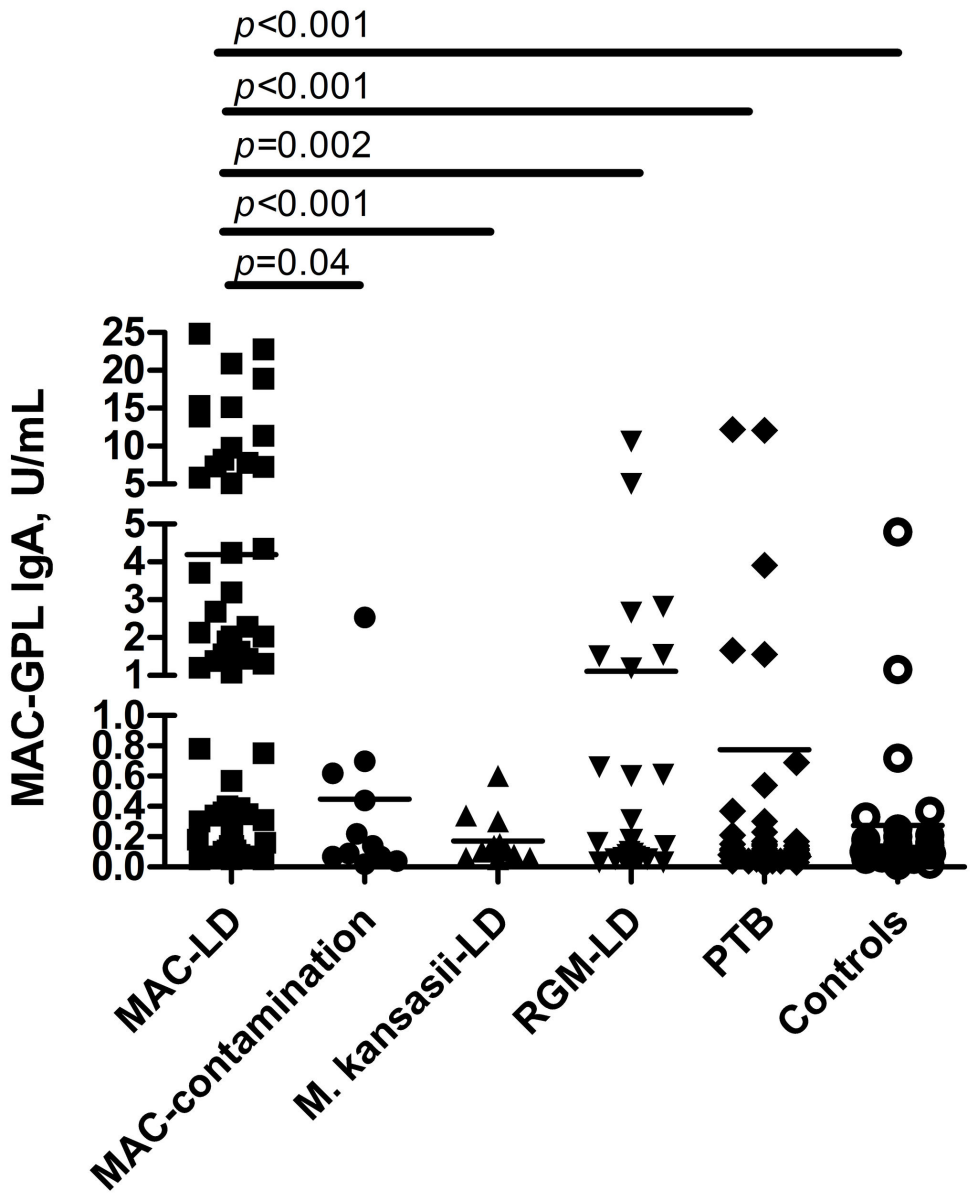
Serum immunoglobulin A antibody levels of Mycobacterium avium complex (MAC) glycopeptidolipid (GPL) in different lung disease statuses. IgA, immunoglobulin A; LD, lung disease; PTB, pulmonary tuberculosis; RGM, rapid-growing mycobacteria.

Regarding MAC-GPL IgA, MAC-LD patients had significantly higher serum levels than all of the other groups (Figure 1). Multivariate logistic regression revealed that age (odds ratio [OR]: 1.032; 95% CI: 1.005-1.060), presence of autoimmune disease (OR: 4.884; 95% CI: 1.038-22.971), and serum MAC-GPL IgA antibody level (OR: 1.325; 95% CI: 1.144-1.536) were independent predictors of MAC-LD (Table 2). As regards patients with respiratory specimen(s) culture-positive for MAC, only MAC-GPL IgA level (OR: 2.856; 95% CI: 1.045-7.805) remained a significant factor for differentiating MAC-LD from MAC contamination. 

**Table 2:** Factors associated with Mycobacterium avium complex-lung disease (MAC-LD), by multivariate logistic regression.

**Characteristics**	**Multivariate**	
	***p* value**	**OR (95% C.I.)**
Age, per year increment	0.020	1.032 (1.005 ~ 1.060)
Sex, male	0.204	
Presence of underlying co-morbidity	0.341	
Malignancy	0.921	
Diabetes mellitus	0.270	
Liver cirrhosis	0.339	
Hemodialysis (chronic renal failure)	0.999	
Autoimmune disease	0.045	4.884 (1.038 ~ 22.971)
Blood leukocyte count, per L	0.236	
Hemoglobin, per g/dL	0.335	
MAC GPL antibody, per U/mL increment	<0.001	1.325 (1.144 ~ 1.536)

Abbreviation: GPL, glycopeptidolipid

**Figure 2:**
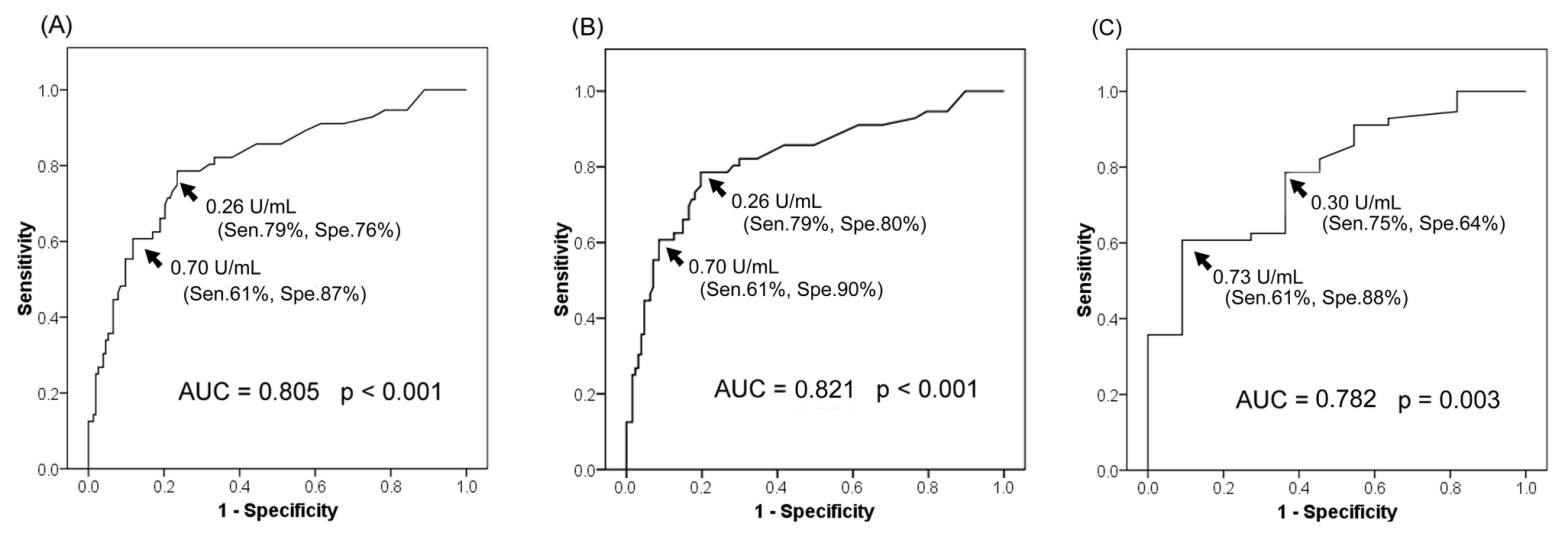
The receiver operating characteristic (ROC) curves were plotted for predicting patients with Mycobacterium avium complex (MAC)-lung disease in different population. (**A**) all participants, (**B**) all except RGM-LD patients, and (**C**) those with positive sputum culture for MAC. The numbers in parenthesis represent the corresponding sensitivity and specificity using the specific cut-off value. AUC, area under curve; Sen, sensitivity; Spe, specificity.

The ROC curve analysis revealed that serum MAC-GPL IgA had high discriminative power for MAC-LD from all other groups (AUC 0.805, *p*<0.001) (Figure 2A), all other groups except RGM-LD (AUC 0.821, *p*<0.001) (Figure 2B), or MAC contamination (AUC 0.782; *p*=0.003) (Figure 2C). For discriminating MAC-LD from all other groups, the optimal cut-off value of serum MAC-GPL IgA was 0.26 U/ml (sensitivity 79%; specificity 76%). If the cut-off value was increased to 0.7 U/mL by the manufacturer's instruction, sensitivity and specificity became 61% and 87%, respectively. 

For discriminating MAC-LD from all other groups except RGM-LD, the optimal cut-off value of MAC-GPL IgA was 0.26 U/ml (sensitivity 79%; specificity 80%). The optimal cut-off value of MAC-GPL IgA to discriminate MAC-LD from MAC contamination was 0.73 U/mL (sensitivity 60%; specificity 91%). If the cut-off value was lowered from 0.73 to 0.30 U/mL, sensitivity increased to 75% but specificity decreased to 64%. Using the cut-off value of 0.73 U/ml for discriminating MAC-LD from all other groups, sensitivity was 61% and specificity 88%. 

The proportion of sero-positive cases using 0.73 U/ml as the cut-off value in each group was shown in Table 3. Seven (27%) RGM-LD patients had positive MAC-GPL IgA test. The false-positive rate in RGM-LD patients was significantly higher than that in *M. kansasii*-LD patients (*p*=0.025) and TB household contacts (*p*=0.033), and borderline higher than that in pulmonary TB (*p*=0.066) patients. None of the IGRA-positive TB household contacts had a positive result of the MAC-GPL IgA, whereas two (11%) of the 18 IGRA-negative contacts without underlying disease did.

**Table 3:** Number and proportion of sero-positive subjects in each group based on the cut-off value of 0.73 U/ml for *Mycobacterium avium* complex glycopeptidolipid antibody.

Clinical diagnosis	Sero-positive, n (%)
MAC-LD (n=57)	34 (60%)
MAC contamination (n=11)	1 (9%)
*M. kansasii*-LD (n=13)	0
RGM-LD (n=26)	7 (27%)
Pulmonary TB (n=48)	5 (10%)
TB household contacts (n=42)	2 (5%)

Abbreviations: LD, lung disease; MAC, *Mycobacterium avium* complex; RGM, rapid-growing mycobacteria; TB, tuberculosis

In MAC-LD, patients with co-morbidity had lower MAC-GPL antibody levels than those without (1.50 vs. 5.54 IU/ml, *p*=0.009) and patients with malignancy had lower serum GPL IgA levels than those without (0.40 vs. 4.70 IU/ml, *p*=0.018). Within the MAC-LD group, patients with high smear grade had higher serum MAC-GPL IgA levels than those with low smear grade and negative smear (7.89 vs. 3.41 IU/mL, *p*=0.039). The MAC-GPL IgA serum level was borderline higher in 46 MAC-LD patients with ≥2 positive sputum cultures for MAC within six months before blood sampling, compared to the other 10 MAC-LD patients (4.76 vs. 2.01 U/mL, *p*=0.072). The ROC curve predicting those with ≥2 sputum cultures positive for MAC within six months from all participants with any sputum positive for MAC revealed that serum MAC-GPL IgA had good discriminative power (AUC 0.728, *p*=0.005) with the optimal cut-off value of 0.73 U/mL (sensitivity 63%; specificity 78%). 

The median MAC-GPL IgA serum level was similar between those with and without prior TB history (1.32 vs. 1.47 U/ml, *p* = 0.982 by Mann-Whitney *U* test). The number of positive culture, disease duration before blood sampling, started NTM treatment, and radiographic pattern were not significantly correlated with MAC-GPL IgA level. Among the 56 MAC-LD patients, the average MAC-GPL IgA was similar in the 10 with bronchoscopic specimen(s) positive for MAC and the other 46 without (4.11 vs. 4.99 U/mL, *p*=0.694). 

In multivariate analysis for MAC-GPL IgA ≥0.73 U/ml in MAC-LD, the presence of co-morbidity was the only independent factor (OR: 0.218; 95% CI: 0.063-0.754) (*p*=0.016) whereas positive culture for MAC ≥3 sets was a borderline predictor (OR: 3.530; 95% CI: 0.866-14.395) (*p*=0.079).

## Discussion

In the present study, serum MAC-GPL IgA level is higher in MAC-LD than MAC contamination and other mycobacterial lung diseases. Using a cut-off value of 0.73 U/ml leads to an intermediate sensitivity but excellent specificity for identifying patients with MAC-LD. In MAC-LD, immuno-competence is independently associated with higher serum MAC-GPL IgA level.

Inflammatory markers like C-reactive protein, procalcitonin, and interferon-gamma are poorly associated with NTM-LD. Soluble triggering receptors expressed on myeloid cell-1 have better correlation with NTM-LD though these are not pathognomonic [7]. In contrast, MAC-GPL antibody is more specific to MAC-LD and has been developed since the last decade [9,10]. Such GPL belong to a class of glycolipids produced by several NTM [15,16] and is an important element in NTM cell wall. GPL is located in the outmost layer, and is thus very antigenic [16]. A previous study conducted in a Japanese population reported 100% specificity and 84% sensitivity in differentiating MAC-LD from other lung disease using a cut-off value of 0.7 U/ml for MAC-GPL IgA serum level [10]. Such finding suggests that GPL of MAC is antigenic and its antibody maybe a good diagnostic marker. 

However, in a study conducted in the United States, the sensitivity of MAC-GPL IgA for diagnosing MAC-LD from contamination is only 51.7% and 70.1% using cut-off values of 0.7 and 0.3 U/ml, respectively [11]. Similarly, the present study shows that MAC-GPL IgA has an intermediate sensitivity but an excellent specificity for discriminating MAC-LD using a cut-off value of 0.73 U/ml. It is not sensitive enough to be diagnostic for MAC-LD in clinically suspected cases because of the high false-negative rate. 

The sub-optimal sensitivity of the MAC-GPL IgA test may be explained by two reasons. First, previous studies mainly included immunocompetent patients but 32% of the MAC-LD patients in this study had some degree of immuno-compromise, which was associated with lower GPL antibody. The finding is similar to the study conducted in the US wherein 21% of the enrolled subjects were given immuno-suppressants. In the MAC-LD patients here, immuno-compromised disease such as malignancy was a major factor associated with low MAC-GPL IgA serum level. The average MAC-GPL IgA in the present study was around 4.3 U/ml, far less than the 10.7 U/ml reported in the study in Japan. Because immuno-compromised hosts have a higher probability of MAC-LD, further large-scale prospective studies are required to evaluate diagnostic performance in a population with different co-morbidity. Second, the interval of sputum positivity may matter. The MAC-LD patients without ≥2 positive sputum culture within the last six months had lower MAC-GPL IgA level, suggesting that proper timing after sputum positivity might be needed to apply this sero-diagnostic test. 

The discriminative power of MAC-GPL IgA test between MAC-LD and RGM-LD is poor. The seropositivity rate is around 27% in diseases due to RGM, probably because GPL is also a cell wall component in RGM [17]. Since culture turn-around-time for RGM is usually less than one week [1], the diagnosis of RGM-LD can possibly be immediately excluded in suspected cases of MAC-LD. The specificity of MAC-GPL IgA test, therefore, increases if patients with RGM-LD are excluded. For the other groups, the false-positive rate is ≤10%, which is comparable to those of previous reports [9,11]. This may be explained by the immune response due to prior or asymptomatic MAC infection [18]. Given the possible existence of false-positive results, MAC-GPL IgA is not a suitable diagnostic test for MAC-LD before the initial mycobacterial data becomes available. 

For a case of MAC contamination that is sero-positive for MAC-GPL IgA antibody, MAC-LD may be underestimated because of the low sensitivity of the ATS diagnostic criteria, especially when the patient is unable to produce/expectorate adequate sputum. However, anti-MAC chemotherapy is not recommended until the diagnosis of MAC-LD has been confirmed due to the possibility of adverse events [1,19]. The serologic test for MAC-GPL IgA may thus be useful for patients who have a respiratory sample culture-positive for MAC but in whom invasive diagnostic procedures may be risky.

The present study has some limitations. First, the case number studied is small, precluding a firm conclusion on the clinical usefulness of the MAC-GPL IgA test. The diagnostic strategy for each sub-group with different underlying diseases should also be proven by a large-scale study in the future. Second, since persons with MAC contamination are usually asymptomatic, enrolling them into the study is much more difficult than enrolling MAC-LD patients. Therefore, the number of cases of MAC contamination is particularly small. Third, although TB household contacts were free of active lung disease, they may have been exposed to MAC. Lastly, because the study was conducted in a tertiary referral center, the prevalence of co-morbidity was high, thus limiting the external generalization of the study. 

In conclusion, serum level of MAC-GPL IgA antibody is higher in MAC-LD than MAC contamination and other lung diseases. Using a cut-off value of 0.73 U/mL leads to an intermediate sensitivity (60%) but good specificity (91%) for differentiating MAC-LD from MAC contamination. This might be helpful in suggesting pulmonary infection by MAC when a respiratory specimen was culture-positive for MAC but further invasive diagnostic procedure like bronchoscopy is risky. Careful consideration is needed while using MAC-GPL IgA antibody in immuno-compromised hosts. 

## References

[1] Griffith DE, Aksamit T, Brown-Elliott BA, Catanzaro A, Daley C et al. (2007) An official ATS/IDSA statement: diagnosis, treatment, and prevention of nontuberculous mycobacterial diseases. Am J Respir Crit Care Med 175: 367-416. doi:10.1164/rccm.200604-571ST. PubMed: 17277290. 17277290

[2] Lai CC, Tan CK, Chou CH, Hsu HL, Liao CH et al. (2010) Increasing incidence of nontuberculous mycobacteria, Taiwan, 2000-2008. Emerg Infect Dis 16: 294-296. doi:10.3201/eid1602.090675. PubMed: 20113563. 20113563 PMC2958002

[3] Shu CC, Lee CH, Hsu CL, Wang JT, Wang JY et al. (2011) Clinical characteristics and prognosis of nontuberculous mycobacterial lung disease with different radiographic patterns. Lung 189: 467-474. doi:10.1007/s00408-011-9321-4. PubMed: 21956280. 21956280

[4] Field SK, Cowie RL (2006) Lung disease due to the more common nontuberculous mycobacteria. Chest 129: 1653-1672. doi:10.1378/chest.129.6.1653. PubMed: 16778288. 16778288

[5] Koh WJ, Kwon OJ, Jeon K, Kim TS, Lee KS et al. (2006) Clinical significance of nontuberculous mycobacteria isolated from respiratory specimens in Korea. Chest 129: 341-348. doi:10.1378/chest.129.2.341. PubMed: 16478850. 16478850

[6] van Ingen J, Bendien SA, de Lange WC, Hoefsloot W, Dekhuijzen PN et al. (2009) Clinical relevance of non-tuberculous mycobacteria isolated in the Nijmegen-Arnhem region, The Netherlands. Thorax 64: 502-506. doi:10.1136/thx.2008.110957. PubMed: 19213773. 19213773

[7] Shu CC, Lee LN, Wu MF, Lee CH, Wang JT et al. (2011) Use of soluble triggering receptor expressed on myeloid cells-1 in non-tuberculous mycobacterial lung disease. Int J Tuberc Lung Dis 15: 1415-1420. doi:10.5588/ijtld.10.0786. PubMed: 22283904. 22283904

[8] Shu CC, Lee CH, Wang JY, Jerng JS, Yu CJ et al. (2008) Nontuberculous mycobacteria pulmonary infection in medical intensive care unit: the incidence, patient characteristics, and clinical significance. Intensive Care Med 34: 2194-2201. doi:10.1007/s00134-008-1221-6. PubMed: 18648768. 18648768

[9] Kitada S, Maekura R, Toyoshima N, Fujiwara N, Yano I et al. (2002) Serodiagnosis of pulmonary disease due to Mycobacterium avium complex with an enzyme immunoassay that uses a mixture of glycopeptidolipid antigens. Clin Infect Dis 35: 1328-1335. doi:10.1086/344277. PubMed: 12439795. 12439795

[10] Kitada S, Kobayashi K, Ichiyama S, Takakura S, Sakatani M et al. (2008) Serodiagnosis of Mycobacterium avium-complex pulmonary disease using an enzyme immunoassay kit. Am J Respir Crit Care Med 177: 793-797. doi:10.1164/rccm.200705-771OC. PubMed: 18079497. 18079497

[11] Kitada S, Levin A, Hiserote M, Harbeck RJ, Czaja CA et al. (2013) Serodiagnosis of Mycobacterium avium complex pulmonary disease in the United States. Eur Respir J 42: 454-460. doi:10.1183/09031936.00098212. PubMed: 23100506. 23100506

[12] Wang JY, Shu CC, Lee CH, Yu CJ, Lee LN et al. (2012) Interferon-gamma release assay and Rifampicin therapy for household contacts of tuberculosis. J Infect 64: 291-298. doi:10.1016/j.jinf.2011.11.028. PubMed: 22207002. 22207002

[13] Wang JY, Hsueh PR, Jan IS, Lee LN, Liaw YS et al. (2006) Empirical treatment with a fluoroquinolone delays the treatment for tuberculosis and is associated with a poor prognosis in endemic areas. Thorax 61: 903-908. doi:10.1136/thx.2005.056887. PubMed: 16809417. 16809417 PMC2104756

[14] Lin JW, Chang YC, Li HY, Chien YF, Wu MY et al. (2009) Cross-sectional validation of diabetes risk scores for predicting diabetes, metabolic syndrome, and chronic kidney disease in Taiwanese. Diabetes Care 32: 2294-2296. doi:10.2337/dc09-0694. PubMed: 19755627. 19755627 PMC2782993

[15] Ripoll F, Deshayes C, Pasek S, Laval F, Beretti JL et al. (2007) Genomics of glycopeptidolipid biosynthesis in Mycobacterium abscessus and M. chelonae. BMC Genomics 8: 114. doi:10.1186/1471-2164-8-114. PubMed: 17490474. 17490474 PMC1885439

[16] Schorey JS, Sweet L (2008) The mycobacterial glycopeptidolipids: structure, function, and their role in pathogenesis. Glycobiology 18: 832-841. doi:10.1093/glycob/cwn076. PubMed: 18723691. 18723691 PMC2733780

[17] Chatterjee D, Khoo KH (2001) The surface glycopeptidolipids of mycobacteria: structures and biological properties. Cell Mol Life Sci 58: 2018-2042. doi:10.1007/PL00000834. PubMed: 11814054. 11814054 PMC11337330

[18] Khan K, Wang J, Marras TK (2007) Nontuberculous mycobacterial sensitization in the United States: national trends over three decades. Am J Respir Crit Care Med 176: 306-313. doi:10.1164/rccm.200702-201OC. PubMed: 17507546. 17507546

[19] Tanaka E, Kimoto T, Tsuyuguchi K, Watanabe I, Matsumoto H et al. (1999) Effect of clarithromycin regimen for Mycobacterium avium complex pulmonary disease. Am J Respir Crit Care Med 160: 866-872. doi:10.1164/ajrccm.160.3.9811086. PubMed: 10471610. 10471610

